# Aligning evidence: concerns regarding multiple sequence alignments in estimating the phylogeny of the Nudibranchia suborder Doridina

**DOI:** 10.1098/rsos.171095

**Published:** 2017-10-25

**Authors:** Joshua M. Hallas, Anton Chichvarkhin, Terrence M. Gosliner

**Affiliations:** 1 Department of Biology, University of Nevada, Reno. 1664 N. Virginia St, Reno, NV 89557, USA; 2 Department of Invertebrate Zoology and Geology, California Academy of Sciences, 55 Music Concourse Dr Golden Gate Park, San Francisco, CA 94118, USA; 3 National Scientific Center of Marine Biology, Far East Branch of Russian Academy of Sciences, Palchevskogo 17, Vladivostok 690041, Russia; 4 Far Eastern Federal University, Sukhanova 8, Vladivostok 690950, Russia

**Keywords:** sequence homology, multiple sequence alignment, Dorid systematics, Phanerobranchia, Cryptobranchia

## Abstract

Molecular estimates of phylogenetic relationships rely heavily on multiple sequence alignment construction. There has been little consensus, however, on how to properly address issues pertaining to the alignment of variable regions. Here, we construct alignments from four commonly sequenced molecular markers (16S, 18S, 28S and cytochrome c oxidase subunit I) for the Nudibranchia using three different methodologies: (i) strict mathematical algorithm; (ii) exclusion of variable or divergent regions and (iii) manually curated, and examine how different alignment construction methods can affect phylogenetic signal and phylogenetic estimates for the suborder Doridina. Phylogenetic informativeness (PI) profiles suggest that the molecular markers tested lack the power to resolve relationships at the base of the Doridina, while being more robust at family-level classifications. This supports the lack of consistent resolution between the 19 families within the Doridina across all three alignments. Most of the 19 families were recovered as monophyletic, and instances of non-monophyletic families were consistently recovered between analyses. We conclude that the alignment of variable regions has some effect on phylogenetic estimates of the Doridina, but these effects can vary depending on the size and scope of the phylogenetic query and PI of molecular markers.

## 1. Introduction

The debate regarding multiple sequence alignment (MSA) construction [1–8] and molecular marker selection [9] for use in phylogenetic estimates has been well established in the literature. There is little consensus, however, on the most accurate and replicable approach when considering MSA assemblies [10]. The crux of the matter is how best to align and represent homology in highly variable regions of sequence data. Most approaches employ a combination of a mathematical algorithm and manual curation of variable regions. Unfortunately, in most instances manual curation is loosely defined—which makes subsequent studies difficult to replicate—and even though mathematical algorithms allow for studies to be easily replicated, they do not take into account evolutionary history and consider homology exclusively as similarity [3]. In addition, they often fail to accurately align variable regions [11].

The estimation of evolutionary relationships relies heavily on the ability to determine homology between any given set of characters. The wrongful designation of characters as homologous can confound interpretations of evolutionary relationships by creating homoplasies, which results in the loss of phylogenetic signal [12]. Thus, the determination of homology in either morphological character matrices or MSAs (computational or manually curated) has a direct impact on the accuracy and ability to replicate phylogenetic estimates. This has led to differing positions concerning variable regions and their importance in increasing [13,14] or decreasing [15–17] phylogenetic signal. These highly variable loop regions become increasingly more difficult to align as sequences become more divergent. This confounds the ability to determine accuracy in MSAs and may result in contradictory estimates of deeper evolutionary relationships. It has been proposed that the large sections created by highly variable loop regions in MSAs should be excluded because of the uncertainty in determining reliable estimates of positional homology [18]. Conversely, it has also been suggested that effects from MSAs should be explored in determining phylogenetic estimates due to their likelihood of containing phylogenetic signal [19,20].

Highly divergent sequences and a general lack of diverse molecular markers have made MSAs and resulting phylogenetic estimates for the Nudibranchia problematic [21–26]. For these reasons, we examine how MSA construction of common nDNA and mtDNA markers affect phylogenetic estimates of the diverse Nudibranchia clade Doridina. Dorid nudibranchs have been a robust model for investigating biochemical diversity [27–33], morphological evolution [34–42], colour evolution [43–49], population structure [50–52] and development [53–57]. Even though there have been previous phylogenetic estimates that included dorid nudibranchs (e.g. [35,36]), they did not include representatives of all 19 families within the Doridina, and thus, a comprehensive phylogenetic context for the evolution of these traits is deficient.

The Doridina suborder currently consists of 19 families and more than 2000 described species. The classification has been divided into five superfamilies (Bathydoridoidea, Doridoidea, Onchidoridoidea, Phyllidioidea and Polyceroidea) defined by morphological variation in gill and feeding structures [21,36,58,59]. Species that possess the ability to retract their gills into fully formed gill pockets, Eudoridoidea (=Cryptobranchia), are divided into two clades based on the presence or absence of radula, Labiostomata (=Doridoidea) and Porostomata (=Phyllidiodea), respectively [36]. Dorid nudibranchs that lack a fully formed gill pocket represent the Anthobranchia (=Phanerobranchia), which is subdivided into the Suctoria (=Onchidoridoidea) and the Non-Suctoria (=Polyceroidea) based on the presence or absence of a buccal pump. Even though morphological [36,59] and molecular [22,23,60,61] analyses have shown the Doridina to be monophyletic, no study has focused on a complete sampling of all currently recognized families. Thus, relationships between families are poorly understood due to conflicting phylogenies and inadequate sampling.

In this study, we use three common MSA methodologies: (i) strict mathematical algorithm; (ii) exclusion of variable or divergent regions and (iii) manually curated. We then discuss issues pertaining to the lack of resolution in phylogenetic estimates and limitations in resolving the base of the Doridina. We also address the possible effects alignment construction may have on phylogenetic informativeness (PI), and how phylogenetic signal may shift given alignment methodology. Lastly, we re-evaluate taxonomic classifications that were consistent between all three analyses.

## 2. Material and methods

### 2.1. Taxon sampling

We sampled (121 taxa) representatives from the five superfamilies and all 19 families that currently comprise the Doridina, and mined GenBank for specimens that had at least two sequences. To limit the amount of missing data, we obtained extractions or tissue samples from published specimens to sequence molecular markers that were previously unattained. *Pleurobranchaea meckeli* and *Berthella martensi* were chosen as outgroups because the Pleurobranchomorpha has been suggested to be sister to the Nudibranchia [23]. Additional members from the Aeolidina and Dendronotina were also used in testing the monophyly of the Doridina. Specimens, voucher numbers, collection sites and GenBank accession numbers are listed in table 1. Voucher specimens are located in the collections at California Academy of Sciences (CASIZ) and the Museum of National Scientific Center of Marine Biology, Vladivostok (MIMB).

**Table 1.** Specimens successfully sequenced and used for molecular analyses. Voucher numbers, GenBank accession numbers and collection localities.

		GenBank accession number				
specimen	voucher	16S	18S	28S	COI	locality
PLEUROBRANCHOIDEA						
*Pleurobranchaea meckeli* (Blainville, 1825)		FJ917439	FJ917449	FJ917481	FJ917499	Mediterranean Sea, Spain
Pleurobranchidae						
* Berthella martensi* (Pilsbry, 1896)	MZUCR 6982	HM162592	MF958319	MF958363	HM162683	Las Secas, Islas sin nombre, Panama
AEOLIDINA						
Flabellinidae						
* Flabellina pedata* (Montagu, 1815)		AF249247	AJ224788	—	AF249817	North Sea, Helgoland
DENDRONOTINA						
Arminidae						
* Armina loveni* (Bergh, 1866)		AF249243	AF249196	—	AF249781	North Sea, Kattegat
DORIDINA						
Bathydoridoidea						
Bathydorididae						
* Prodoris clavigera* (Thiele, 1912)	CASIZ 167553	JX274067	MF958320	MF958364	JX274106	Elephant Island, Antarctica
Doridoidea						
Actinocyclidae						
* Actinocyclus verrucosus* Ehrenberg, 1831	CASIZ 189448	MF958311	MF958352	MF958397	MF958438	Kauai, Hawaii
* Hallaxa indecora* (Bergh, 1905)	CASIZ 179600	MF958302	MF958340	MF958386	—	South Loi Island, Marshall Islands
* Hallaxa translucens* Gosliner & S. Johnson, 1994	CASIZ 173447	EU982814	MF958341	MF958387	EU982760	Iles Radama, Madagascar
* Hallaxa iju* Gosliner & S. Johnson, 1994	CASIZ 175559	EU982813	—	—	EU982759	Maui, Hawaii
Cadlinidae						
* Aldisa sanguinea* (J. G. Cooper, 1863)	CASIZ 182031	MF958309	MF958350	MF958394	MF958435	Marin Co., California
* Aldisa* sp.	CASIZ 175733	EU982818	MF958351	MF958395	MF958436	Tiger Reef, Malaysia
* Cadlina cf. luteomarginata* (MacFarland, 1905)	CASIZ 188599A	KJ653679	KP340317	KP340350	KM219678	Parksville, Vancouver Island, British Columbia
* Cadlina laevis* (Linnaeus, 1767)	CASIZ 175444	—	MF958359	MF958406	—	Scotland
* Cadlina luarna* (Marcus & Marcus, 1967)	CASIZ 175437	EU982768	—	—	EU982718	Guanacaste, Costa Rica
* Cadlina modesta* MacFarland, 1966	CASIZ 223286	MF958310	—	—	MF958437	Vista del Mar, San Luis Obispo Co., California
* Cadlina pellucida* (Risso, 1826)	CASIZ 175448	EU982774	—	MF958396	EU982724	Ilha de Pessegueiro, Baixo Alentejo Prov., Portugal
* Cadlina sparsa* (Odhner, 1921)	CASIZ 182932	EU982776	—	—	EU982726	La Jolla, San Diego Co., California
Chromodorididae						
* Cadlinella ornatissima* (Risbec, 1928)	CASIZ 177420	MF958284	MF958325	MF958371	MF958415	Maricaban Island, Luzon, Philippines
* Cadlinella ornatissima* (Risbec, 1928)	CASIZ 175452	EU982779	—	—	EU982728	Mooloolaba, Queensland, Australia
* Chromodoris alternata* (Burn, 1957)	SAM D19281	AY458800	EF534031	—	EF535120	Port Phillip Bay, Victoria, Australia
* Chromodoris ambigua* Rudman, 1987	SAM D19260	AY458801	EF534038	—	EF535119	Port Phillip Bay, Victoria, Australia
* Chromodoris quadricolor* (Rüppell & Leuckart, 1830)		AF249241	AJ224773	—	AF249802	Red Sea, Egypt
* Doriprismatica atromarginata* (Cuvier, 1804)		—	AF249211	—	AF249789	Great Barrier Reef, Australia
* 'Felimare' elegans* (Cantraine, 1835)		AF249238	AJ224779	—	AF249787	NE Atlantic, Spain
* 'Felimare' midatlantica* (Gosliner, 1990)	CASIZ 175443	JQ727789	—	—	JQ727898	Islotes do Martinhal, Algarve, Portugal
* 'Felimare' picta verdensis* Ortea, Valds & Garca-Gmez, 1996	CASIZ 179384	HM162594	MF958346	MF958389	HM162685	Gulf of Guinea, Ilha do Principe, Sao Tome & Principe
* 'Felimare' villafranca* (Riaao, 1818)		AF249237	AJ224780	—	—	NE Atlantic, Spain
* 'Felimida' edmundsi* Cervera, Garcia-Gmez & Ortea, 1989	CASIZ 179385	HM162595	MF958347	MF958390	HM162686	Gulf of Guinea, Ilha do Principe, Sao Tome & Principe
* 'Felimida' krohni* (Verany, 1846)		AF249239	AJ224774	AY427445	AF249805	NE Atlantic, Spain
* Goniobranchus geometricus* (Risbec, 1928)	CASIZ 175549	JQ727717	—	—	JQ727842	Nosy Valiha, Iles Radama, Madagascar
* Hypselodoris imperialis* (Pease, 1860)	CASIZ 142952	EU982807	—	—	EU982754	Mala Wharf, Maui, Hawaii
* Hypselodoris infucata* (Rüppell & Leuckart, 1830)		FJ917427	FJ917442	FJ917467	FJ917485	NSW, Australia
* Miamira magnifica* Eliot, 1904	CASIZ 169951	EU982781	—	—	EU982731	Old Woman Island, Queensland, Australia
* Thorunna daniellae* (Kay and Young, 1969)	CASIZ 170055	EU982809	—	—	EU982756	Maalaea Marina, Maui, Hawaii
* Tyrinna evelinae* (Marcus, 1958)	CASIZ 175440	EU982811	—	MF958391	EU982757	Playa Ventana, Guanacaste, Costa Rica
* Tyrinna nobilis* Bergh, 1898	ZSM M20050508	EF534054	EF534035	—	EF535127	
Dorididae						
* Aphelodoris* sp. 1	CASIZ 176920	MF958293	MF958332	MF958379	MF958424	Oudekraal, Cape Prov., South Africa
* Aphelodoris* sp*.* 1		—	GQ326866	—	GQ292033	Auckland, New Zealand
* Aphelodoris luctuosa* (Cheeseman, 1882)		—	GQ326867	—	GQ292042	Auckland, New Zealand
* Doris montereyensis* (Cooper, 1862)	CASIZ 174493	MF958294	MF958333	—	MF958425	Battery Point, Crescent City, Del Norte Co., California
* Doris odhneri* MacFarland, 1966	CASIZ 188014	MF958295	MF958334	MF958380	—	Duxbury Reef, Marin Co., California
* Doris* sp. 8	CASIZ 192348	MF958306	MF958345	—	—	Red Sea, Saudi Arabia
Discodorididae						
* Asteronotus cespitosus* (van Hasselt, 1824)	CASIZ 191096	MF958288	MF958328	MF958375	MF958419	Kranket Island, Papua New Guinea
* Atagema cf osseosa* (Kelaart, 1859)	CASIZ 185142	MF958296	MF958335	—	MF958426	Maui, Hawaii
* Discodoris coerulescens* Bergh, 1888	CASIZ 182850	MF958290	MF958330	MF958377	MF958421	Maricaban Island, Luzon, Philippines
* Halgerda dalanghita* Fahey & Gosliner, 1999	CASIZ 181264	MF958289	MF958329	MF958376	MF958420	Maricaban Island, Luzon, Philippines
Discodorididae						
* Peltodoris nobilis* (MacFarland, 1905)	CASIZ 182223	EU982816	—	—	EU982761	Pillar Point, San Mateo Co., California
* Platydoris sanguinea* Bergh, 1905	CASIZ 177762	MF958285	MF958326	MF958372	MF958416	Maricaban Island, Batangaas Prov., Philippines
* Rostanga calumus* Rudman & Avern, 1989		FJ917427	—	FJ917468	FJ917485	NSW, Australia
* Sclerodoris tuberculata* Eliot, 1904	CASIZ 190788	MF958286	MF958327	MF958373	MF958417	Madang Prov., Papua New Guinea
* Thordisa albomacula* Chan & Gosliner, 2006	CASIZ 179590	MF958287	—	MF958374	MF958418	Kwajalein Atoll, Marshall Islands
ONCHIDORIDOIDEA						
Akiodorididae						
* Armodoris anudeorum* Valdés, Moran & Woods, 2011	LACM 3118	KP340290	GQ326879	KP340355	KP340387	McMurdo Sound, Ross Sea, Antarctica
Calycidorididae						
* Calycidoris guentheri* Abraham, 1876	CASIZ 190966A	KP340301	KP340338	KP340371	KP340397	Chukchi Sea, Alaska
* Diaphorodoris lirulatocauda* Millen, 1985	CASIZ 184341	KP340307	KP340344	KP340377	KP340403	Duxbury Reef, Marin Co., California
* Diaphorodoris luteocincta* (Sars, 1870)	LACM 8.7A	KP340308	—	KP340378	KP340404	Bahia de Algeciras, Cadiz Prov., Spain
* Diaphorodoris cf mitsuii*	CASIZ 185986	KP340310	KP340345	KP340379	KP340406	Sepok Point, Philippines
* Diaphorodoris papillata* Portmann & Sandmeier, 1960	LACM 8.6A	KP340311	—	—	KP340407	Bahia de Algeciras, Cadiz Prov., Spain Peninsula
Corambidae						
* Corambe obscura* (Verrill, 1870)	CASIZ 183942	KP340303	KP340340	KP340373	KP340399	New Castle Portsmouth Bay, New Hampshire
* Corambe pacifica* MacFarland & O'Donoghue, 1929	LACM 2007-2.6C	KP340305	KP340342	KP340375	KP340401	Long Beach Marina, Los Angeles Co., California
* Corambe steinbergae* (Lance, 1962)	CASIZ 190508	KP340306	—	—	KP340402	Pillar Point, San Mateo Co., California
Goniodorididae						
* Ancula gibbsoa* (Risso, 1818)	CASIZ 182028	KP340291	KP340322	KP340356	KP340388	Cumberland Co., Maine
* Ancula gibbsoa* (Risso, 1818)	CASIZ 181211	MF958291	—	—	MF958422	Duxbury Reef, Marin Co., California
* Goniodoris nodosa* (Montagu, 1808)		AF249226	AJ224783	—	AF249788	NE Atlantic, Spain
* Okenia kendi* Gosliner, 2004	CASIZ 186125A	MF958297	—	MF958381	MF958427	Luzon, Philippines
* Trapania reticulata* Rudman, 1987	CASIZ 191431	MF958303	MF958342	—	MF958432	Tab Island, Papua New Guinea
Onchidorididae						
* Acanthodoris atrogriseat*a O'Donoghue, 1927	CASIZ 186000	KJ653646	KP340323	KP340357	KM219646	Puget Sound, Kitsap Co., Washington
* Acanthodoris hudsoni* MacFarland, 1905	CASIZ 179480	KJ653652	KP340324	KP340359	KM219650	Asilomar, Monterey Co., California
* Acanthodoris nanaimoensis* O'Donoghue, 1921	CASIZ 181569A	KJ653656	KP340325	KP340360	KM219657	Pillar Point, San Mateo Co., California
* Acanthodoris pilosa* (Abildgaard, 1789)	CASIZ 183941A	KJ653659	KP340326	KP340361	—	Cobscook Bay, Washington Co., Maine
* Acanthodoris planca* Fahey & Valdés, 2005	CASIZ 176116	KJ653669	KP340327	KP340362	KM219671	Table Bay, Western Cape Prov., South Africa
* Acanthodoris rhodoceras* Cockerell & Eliot, 1905	CASIZ 181572	KJ653671	KP340328	KP340363	KM219673	Pillar Point, San Mateo Co., California
* Knoutsodonta brasiliensis* (Alvim, Padula & Pimenta, 2011)	BNHS-Opistho-336	KC255225	—	—	KC255226	
* Knoutsodonta depressa* (Alder & Hancock, 1842)	CASIZ 186769A	KP340315	KP340347	—	KP340409	Huelva, Spain
* Knoutsodonta jannae* (Millen, 1987)	CASIZ 175578	KP340296	KP340331	KP340366	KP340392	Pillar Point, San Mateo Co., California
* Knoutsodonta oblonga* (Alder & Hancock, 1845)	MN 3010A	—	KP340349	KP340385	KP340410	Mewstone, Skomer, United Kingdom
* Onchidoris bilamellata* (Linnaeus, 1767)	CASIZ 188593	KP340314	KP340346	KP340382	—	Puget Sound, Kitsap Co., Washington
* Onchidoris muricata* Muller, 1776	CASIZ 181312	KJ653676	KP340348	KP340383	KM219680	Asilomar, Monterey Co., California
* Onchidoris evincta* (Millen, 2006)	CASIZ 187758A	KP340293	KP340329	KP340364	KP340390	Puget Sound, Kitsap Co., Washington
* Onchidoris macropompa* Martynov, Korshunova, Sanamyan & Sanamyan, 2009	MIMB 34210	MF958292	MF958331	MF958378	MF958423	Avacha Bay, Kamchatka
* Onchidoris proxima* (Alder & Hancock, 1854)	CASIZ 183921A	KJ653673	KP340336	KP340369	KM219676	Passamaquody Bay Eastport, Washington Co., Maine
* Onchidoris slavi* Martynov, Korshunova, Sanamyan & Sanamyan, 2009	MIMB 34211	—	—	MF958409	MF958446	Avacha Bay, Kamchatka
* Onchimira cavifer* Martynov, Korshunova, Sanamyan & Sanamyan, 2009	MIMB 34209	MF958298	MF958336	MF958382	MF958428	Avacha Bay, Kamchatka
* Onchimira cavifer* Martynov, Korshunova, Sanamyan & Sanamyan, 2009		—	MF958360	MF958407	MF958445	Avacha Bay, Kamchatka
PHYLLIDIOIDEA						
Dendrodorididae						
* Dendrodoris arborescens* (Collingwood, 1881)	CMNH-ZM08965	—	AB917459	—	AB917436	Oka, Tateyama
* Dendrodoris atromaculata* (Alder & Hancock, 1864)	CASIZ 181231	MF958307	MF958348	MF958392	MF958434	Janao Bay, Luzon, Philippines
* Dendrodoris denisoni* (Angas, 1864)	CASIZ 177702	MF958308	MF958349	MF958393	—	Janao Bay, Luzon, Philippines
* Dendrodoris fumata* (Rüppell & Leuckart, 1830)	CASIZ 192304	—	MF958358	MF958405	MF958444	Red Sea, Saudi Arabia
* Dendrodoris fumata* (Rüppell & Leuckart, 1830)		—	AF249216	FJ917470	AF249799	Great Barrier Reef, Australia
* Dendrodoris guttata* (Odhner, 1917)	CMNH-ZM 08967	—	AB917461	—	AB917446	Okinoshima, Tateyama
* Dendrodoris nigra* (Stimpson, 1855)	CASIZ 182821	MF958318	MF958357	MF958404	MF958443	Maricaban Island, Luzon, Philippines
* Dendrodoris nigra* (Stimpson, 1855)		AF249242	AF249215	—	AF249795	Great Barrier Reef, Australia
* Doriopsilla albopunctata* (J. G. Cooper, 1863)	CPIC 00909	KR002428	—	—	KR002480	Long Beach, California
* Doriopsilla bertschi* Hoover, Lindsay, Goddard & Valdés, 2015	CPIC 01058	KR002462	—	—	KR002517	Bahía de los Ángeles, Baja California, Mexico
* Doriopsilla davebehrensi* Hoover, Lindsay, Goddard & Valdés, 2015	LACM 3419	KR002476	—	—	—	Bahía de los Ángeles, Baja California, Mexico
* Doriopsilla fulva* (MacFarland, 1905)	CPIC 00933	KR002444	—	—	KR002498	Malibu, California
* Doriopsilla gemela* Gosliner, Schaefer & Millen, 1999	CPIC 00938	KR002453	—	—	KR002506	Malibu, California
* Doriopsilla janaina* Marcus &. Marcus, 1967	CASIZ 173618	MF958312	MF958353	MF958398	—	Galápagos Islands, Ecuador
* Doriopsilla miniata* (Alder & Hancock, 1864)	CMNH ZM08970	—	AB917464	—	AB917457	Yoshio, Katsuura
* Doriopsilla spaldingi* Valdés & Behrens, 1998	CPIC 00908	KR002427	—	—	KR002479	San Pedro, Los Angele Co., California
Mandeliidae						
* Mandelia mirocornata* Valdés & Gosliner, 1999	CASIZ 176266	MF958278	MF958321	MF958365	MF958411	Oudekraal, Cape Prov., South Africa
Phyllidiidae						
* Ceratophyllidia* sp.	CASIZ 181247	MF958281	MF958323	MF958368	MF958413	Beatrice, Philippines
* Phyllidia coelestis* Bergh, 1905	CASIZ 190982	MF958279	—	MF958366	MF958412	Kranket Island, Madang Prov., Papua New Guinea
* Phyllidiella nigra* (van Hasselt, 1824)	CASIZ 186196A	MF958280	MF958322	MF958367	—	Maricaban Strait, Batangas Prov., Luzon, Philippines
* Phyllidiella pustulosa* (Cuvier, 1804)		AF249232	AF249208	—	—	Great Barrier Reef, Australia
* Phyllidiopsis annae* Brunckhorst, 1993	CASIZ 186138	MF958283	MF958324	MF958370	—	Philippines
* Reticulidia halgerda* Brunckhorst & Burn in Brunckhorst, 1990	CASIZ 186491	MF958282	—	MF958369	MF958414	Maricaban Island, Luzon, Philippines
POLYCEROIDEA						
Aegiridae						
* Aegires albopunctatus* MacFarland, 1905	CASIZ 182213	MF958313	MF958354	MF958399	MF958439	Marin Co., California
* Aegires citrinus* Pruvot-Fol, 1930	CASIZ 144027	MF958314	MF958355	MF958400	MF958440	Mooloolaba, Queensland, Australia
* Aegires flores* Fahey & Gosliner, 2004	CASIZ 191244	MF958316	—	MF958402	MF958442	Papua New Guinea
* Aegires serenae* (Gosliner and Behrens, 1997)	CASIZ 191285	MF958315	—	MF958401	MF958441	Papua New Guinea
* Aegires villosus* Farran, 1905	CASIZ 177563	MF958317	MF958356	MF958403	—	Maricaban Island, Luzon, Philippines
Gymnodorididae						
* Gymnodoris* sp*.*	CASIZ 176781	—	MF958361	—	—	Pulau Penang, Malaysia
Hexabranchiidae						
* Hexabranchus sanguineus* (Ruppell & Leuckart, 1828)	CASIZ 142942	MF958304	MF958343	—	—	Kapalua Bay, Maui, Hawaii
* Hexabranchus sanguineus* (Ruppell & Leuckart, 1828)	CAZIS 193381	MF958305	MF958344	MF958388	MF958433	Papua New Guinea
Okadaiidae						
* Vayssierea* sp.	CASIZ 190731	—	MF958362	MF958408	—	Sunshine Coast, Kings Beach, Australia
Polyceridae						
* Kaloplocamus* sp. 1	CASIZ 194412	MF958299	MF958337	MF958383	MF958429	South Madagascar, Madagascar
* Limacia* sp. 1	CASIZ 176312	HM162602	KP340320	KP340353	HM162692	False Bay, Western Cape Prov., South Africa
* Limacia* sp*.* 2	CASIZ 176276	HM162603	—	—	HM162693	Oudekraal, Cape Prov., South Africa
* Nembrotha cristata* Bergh, 1877	CASIZ 191428	MF958301	MF958339	MF958385	MF958431	Madang Prov., Papua New Guinea
* Plocamopherus pecoso* Valls and Gosliner, 2006	CASIZ 191587	MF958300	MF958338	MF958384	MF958430	Madang Prov., Papua New Guinea
* Polycera quadrilineata* (Müller, 1776)		AF249229	AJ224777	—	—	North Sea, Kattegat
* Roboastra ricei* Pola, Cervera & Gosliner, 2008	CASIZ 173900	HM162598	—	—	HM162688	Florida, 5 mi offshore of Loran Tower
* Tambja marbellensis* Schick & Cervera, 1998	CASIZ 180379	HM162599	—	—	HM162689	Setubal District, Outao, Portugal
* Triopha catalinae* (Cooper, 1863)	CASIZ 170648	HM162600	KP340321	KP340354	HM162690	Yacht Harbor, San Francisco, California
* Triopha maculata* MacFarland, 1905	CASIZ 181556	HM162601	—	—	HM181556	Duxbury Reef, Marin Co., California

### 2.2. Extraction, PCR protocols and sequencing

Genomic DNA was extracted using the Qiagen DNeasy Blood & Tissue extraction kit (Valencia, CA). Amplification and sequencing for targeted gene fragments 16S, 18S, 28S and cytochrome c oxidase subunit I (COI) followed protocols used by Hallas & Gosliner [62]. Amplified fragments were sequenced at the Center for Comparative Genomics located at the California Academy of Sciences and National Scientific Center of Marine Biology.

### 2.3. Multiple sequence alignments

We assembled and edited sequences in Geneious Pro v. 9.1.7 [63] and BioEdit [64]. We aligned rDNA fragments (16S, 18S and 28S) using three different methodologies to examine conflicts regarding estimated phylogenies. For our first method, we used a computer algorithm E-INS-i in MAFFT (MA) [65]. The E-INS-i algorithm is designed to handle sequence data with several conserved regions embedded in long unalignable gapped regions. Our second method excluded all variable regions from the initial MAFFT alignment of rDNA using Gblocks (GA) [11]. We determined blocks using a less stringent selection by allowing for smaller final blocks, gap positions within the final blocks and less strict flank positions. For our third method, we used the initial MAFFT alignment from method one, but manually curated variable regions (CA). This was done by hand to correct for possible inappropriate alignment of sequence regions. For each alignment method, we concatenated all four targeted molecular markers into single MSAs that resulted in three separate concatenated datasets.

### 2.4. Phylogenetic informativeness

We estimated PI for each molecular marker per alignment [9] through the PhyDesign web interface [66], and estimated each PI profile using the site-rates model HyPhy [67]. First, we generated ultrametric trees by converting our concatenated Bayesian inference (BI) phylogenetic estimates in Mesquite [68]. Owing to the lack of a fossil record for the Doridina, our ultrametric trees are not in known time units. However, it has been shown that PI profiles can be used effectively even if divergence time estimates are absent [69]. Then, we analysed alignments in relation to each of their resulting estimated phylogeny to gain a greater understanding of how PI can change based on the alignment approach.

### 2.5. Phylogenetic analyses

We determined evolutionary models using PartitionFinder v.1.1.1 [70], and partitioned our concatenated datasets by rDNA fragment as well as codon position for COI. We also analysed our nDNA and mtDNA fragments separately to investigate possible conflicting evolutionary histories. We did this by aligning both nDNA and mtDNA datasets using the algorithm E-INS-i in MAFFT, and used the same partitioning scheme as in our concatenated datasets.

We analysed all our datasets using BI and maximum-likelihood (ML). BI searchers were run using MrBayes v. 3.2.1 [71], and convergence was checked in TRACER v.1.5 [72]. The datasets were run for 5 × 10^7^ generations with Markov chains sampled every 1000 generations. The standard 25% burn-in was calculated and remaining tree estimates were used to create a 50% majority rule consensus tree of calculated posterior probabilities. Posterior probabilities (pp) that exceeded 0.95 were considered strongly supported, and values 0.94 and below were interpreted as having low support. For our ML analyses, we calculated non-parametric bootstrapping (bs) values and the ML tree simultaneously in RAxML v. 7.2.6 [73]. We used the same partitioning scheme as in our BI search, but used the evolution model GTR+I− and executed fast bootstrapping runs for 5 × 10^4^ iterations. Bootstrap values 70 or higher were considered strongly supported, while all other values were evaluated as weakly supported [74].

## 3. Results

### 3.1. Molecular data

Sequences obtained for phylogenetic analyses and PI profiles are labelled in table 1, and all alignments have been deposited in TreeBASE (http://purl.org/phylo/treebase/phylows/study/TB2:S20396). As expected, our concatenated alignments (MA, GA and CA) varied in length as well as number of parsimony informative characters (table 2). Surprisingly, there were more parsimony informative characters in the MA than the CA. The GA had far fewer parsimony informative characters than the other two alignments generated. Evolutionary model GTR+I+I− was selected by PartitionFinder v. 1.1.1 for each partition based on the Akaike information criterion. Interestingly, specific species of *Dendrodoris* (*D. nigra*, *D. arborescens*, *D. guttata* and *D. fumata*) had mtDNA regions that were highly divergent.

**Table 2.** Summary of multiple sequence alignment variation between alignment methodologies for 16S, 18S, 28S and COI.

	length (base pairs)				parsimony informative characters			
alignment	16S	18S	28S	COI	16S	18S	28S	COI
MAFFT	498	2578	960	661	315	485	388	370
Gblocks	390	1546	585	661	236	351	174	370
Curated	584	2722	1077	661	308	396	324	370

### 3.2. Phylogenetic informativeness

**Figure 1.**
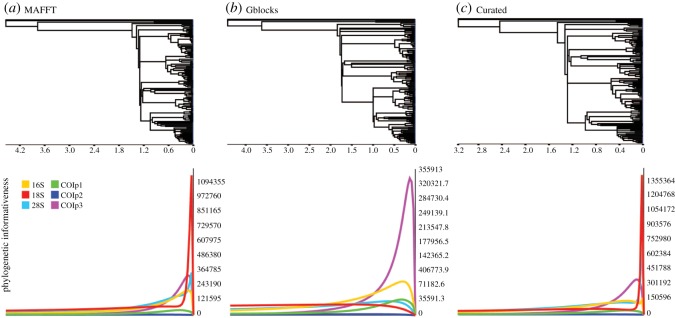
Phylogenetic informativeness profiles estimated by PhyDesign. (*a*) MAFFT alignment. (*b*) Gblocks alignment. (*c*) Curated alignment.

The net PI profiles depict the amount of signal through time of each molecular marker in relation to the respective estimated phylogeny (figure 1*a*–*c*). Regardless of the alignment, net PI profiles show most phylogenetic signal resides towards the tips of the trees. The amount of overall phylogenetic signal was lowest in our GA estimate, which suggests that information was lost when variable regions were excluded. Lastly, our MA appeared to have slightly more PI than our CA.

### 3.3. Phylogeny

**Figure 2.**
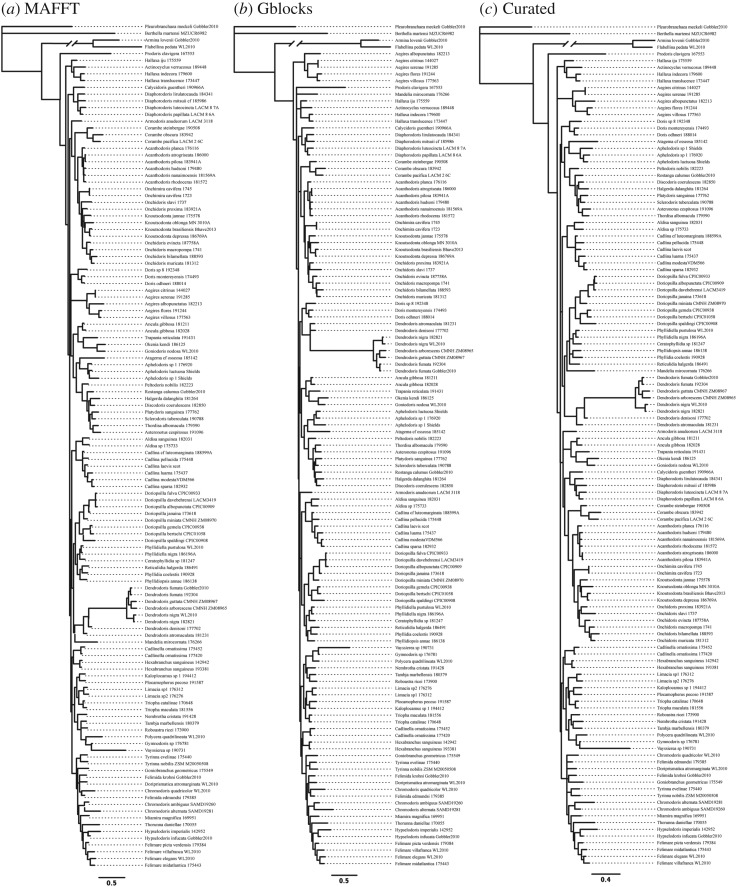
Phylograms of Doridina phylogenetic estimates from all three different alignment constructions. Topologies represent Bayesian estimates. (*a*) MAFFT alignment. (*b*) Gblocks alignment. (*c*) Curated alignment.

**Figure 3.**
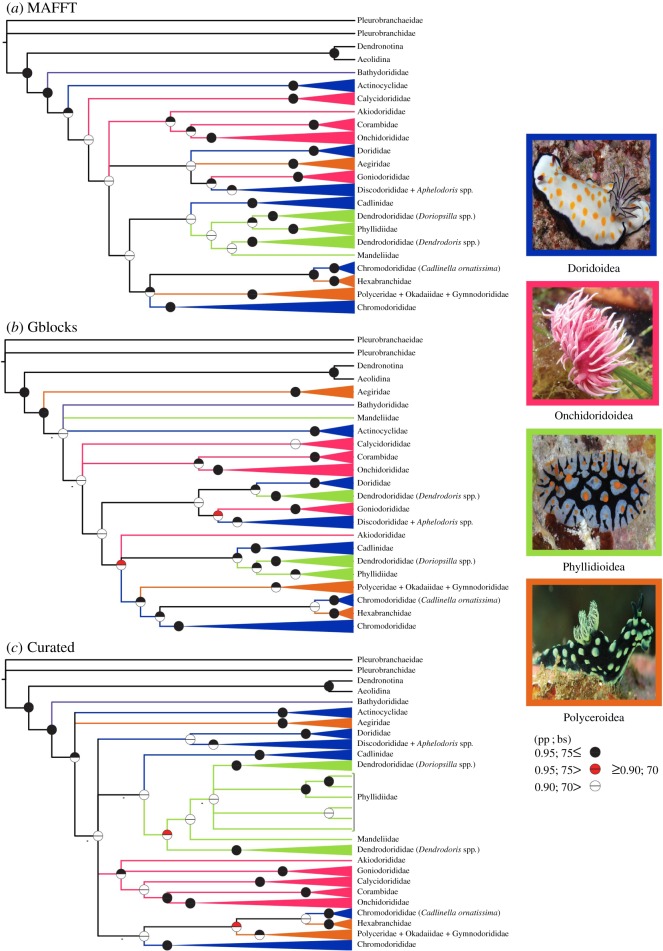
Cladogram of Doridina phylogenetic estimates. Topology represents Bayesian estimate. Branches are coloured based on superfamily designations, which are pictured adjacent to the phylogenies. Only four of the five families are depicted due to the inability to obtain an image of Bathydoridoidea. Circles represent posterior probabilities (top) and non-parametric bootstrap support values (bottom). Closed circles indicate high Bayesian and ML support (pp ≥ 0.95; bs ≥ 75). Red circles indicate moderate support values (pp: 0.95–0.90; bs: 75–70). Open circles indicate no support (pp < 0.90; bs < 70). Relationships that were not recovered by ML analysis are represented by dashes. (*a*) MAFFT alignment. (*b*) Gblocks alignment. (*c*) Curated alignment.

BI posterior probability estimates generally resulted in higher overall support of relationships than ML non-parametric bootstrap values. Branch lengths for basal nodes were extremely short (figure 2*a–c*), especially in regions of the tree that resulted in low resolution for relationships between superfamilies (figure 3*a*–*c*). There were, however, some general similarities between topologies of both phylogenetic searches and among alignments.

**Figure 4.**
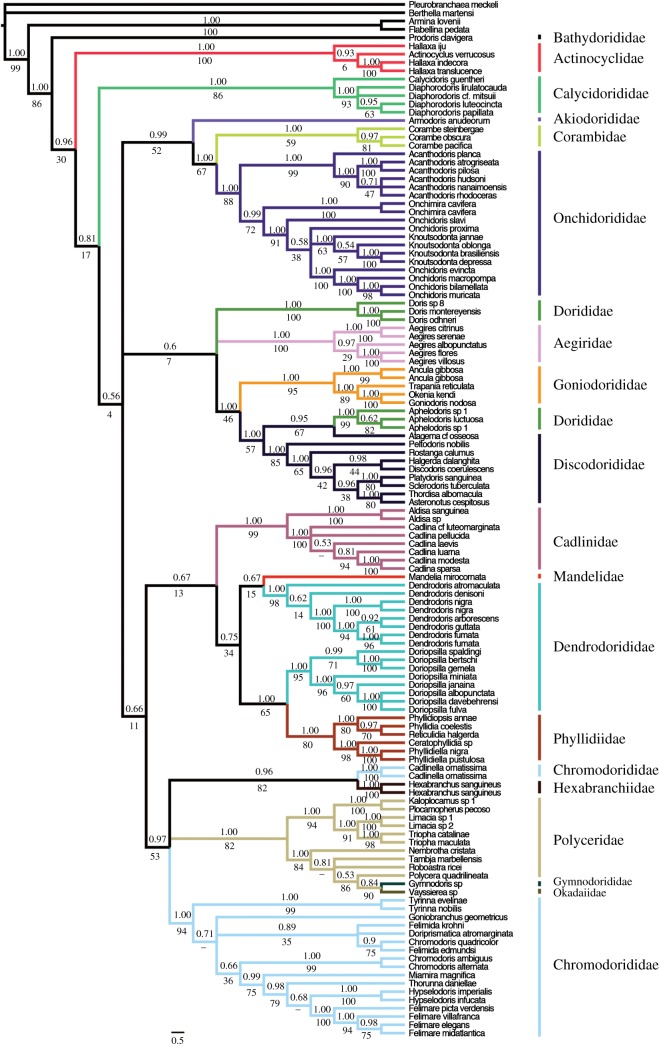
Phylogenetic estimate from MAFFT Alignment. Topology represents Bayesian estimate, with posterior probabilities (pp) and non-parametric bootstrap (bs) support values depicted above and below each branch, respectively. Relationships that were not recovered by ML analysis are represented by dashes. Branches are coloured based on family designations and represented on the exterior of the phylogeny.

**Figure 5.**
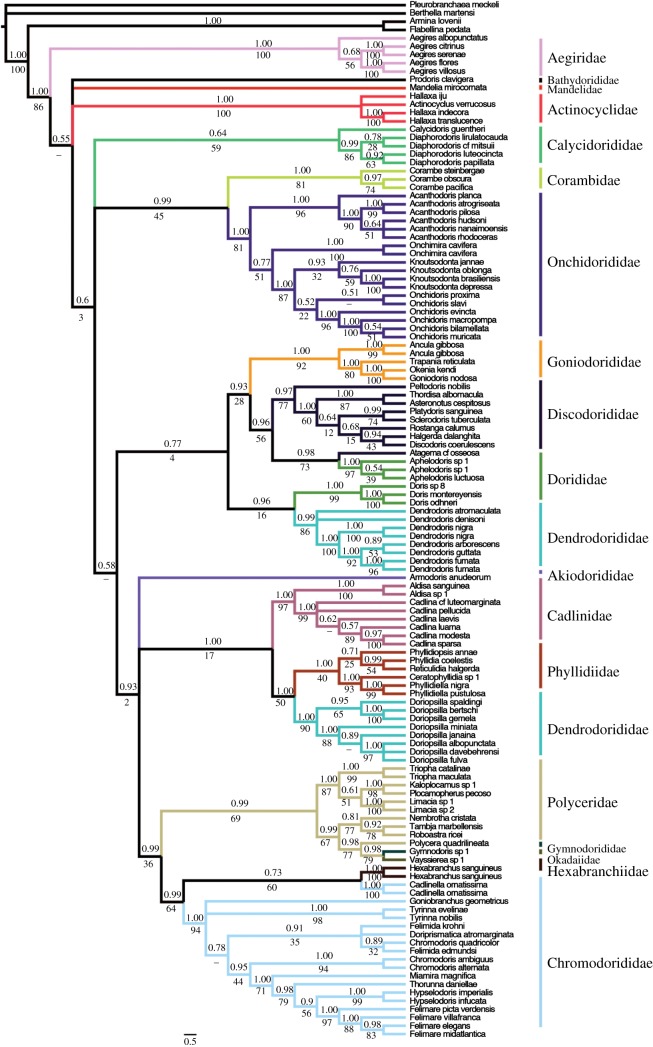
Phylogenetic estimate from Gblocks alignment. Topology represents Bayesian estimate, with posterior probabilities (pp) and non-parametric bootstrap (bs) support values depicted above and below each branch, respectively. Relationships that were not recovered by ML analysis are represented by dashes. Branches are coloured based on family designations and represented on the exterior of the phylogeny.

**Figure 6.**
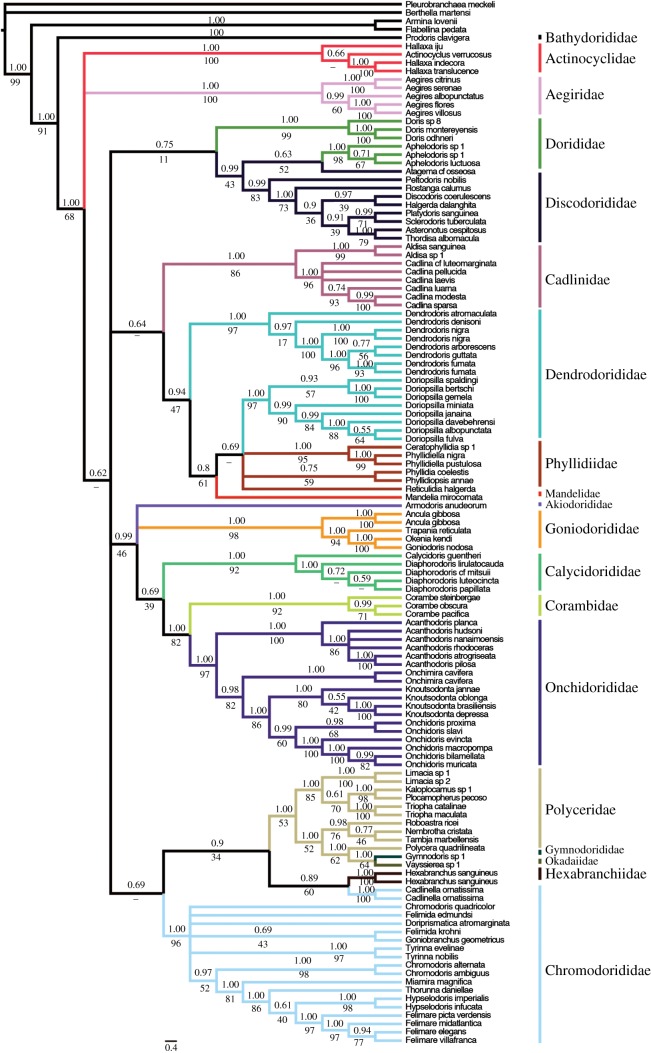
Phylogenetic estimate from Curated alignment. Topology represents Bayesian estimate, with posterior probabilities (pp) and non-parametric bootstrap (bs) support values depicted above and below each branch, respectively. Relationships that were not recovered by ML analysis are represented by dashes. Branches are coloured based on family designations and represented on the exterior of the phylogeny.

The Doridina suborder was recovered as monophyletic with high support in all analyses estimates (CA: pp = 1.00, bs = 91; GA: pp = 1.00, bs = 86; MA: pp = 1.00, bs = 86; figures 4–6). GA was the only analysis that recovered the Aegiridae sister to all other members of the Doridina, but there was no support. Both MA and CA recovered *Prodoris* sister to the rest of the Doridina.

Onchidoridoidea and Phyllidioidea were the only two superfamilies recovered as monophyletic in any of the three analyses (figure 3*a*–*c*). Onchidoridoidea was recovered as monophyletic only in the CA (pp = 0.99, bs = 46). Phyllidioidea was monophyletic with little to no support in CA (pp = 0.94; bs = 47) and MA (pp = 0.75, bs = 34) analyses. Most families, however, were recovered as monophyletic with high pp and bs support across all three alignments analysed. The Phyllidiidae and Calycidorididae were the only two families that were not supported in all three analyses. In addition, Chromodorididae, Dendrodorididae, Dorididae and Polyceridae were consistently recovered as not monophyletic.

The *Doriopsilla* clade was recovered more closely related (GA: pp = 1.00, bs = 50; MA: pp = 1.00, bs = 65) to a monophyletic Phyllidiidae than to the *Dendrodoris* clade. This strongly suggests the Dendrodorididae is not monophyletic. Interestingly, Cadlinidae was recovered as sister to the Phyllidioidea in MA (pp = 0.67, bs = 13) and CA (pp = 0.64), but in our GA estimate Cadlinidae was sister (pp = 1.00, bs = 17) also to a clade formed by Phyllidiidae and *Doriopsilla*. *Aphelodoris* was recovered nested within Discodorididae and sister to *Atagema* cf. *osseosa* (CA: pp = 0.63, bs = 52; GA: pp = 0.98, bs = 73; MA: pp = 0.95, bs = 67). Furthermore, *Vayssierea* sp. and *Gymnodoris* sp*.* were recovered nested in the Polyceridae and sister to *Polycera quadrilineata* (CA: pp = 1.00, bs = 64; GA: pp = 0.98, bs = 77; MA: pp = 0.53, bs = 86).

The Polyceroidea, excluding Aegiridae and Hexabranchidae, was consistently recovered closely related to the Chromodorididae (CA: pp = 0.69; GA: pp = 0.99, bs = 36; MA: pp = 0.97, bs = 53). In addition, all three analyses recovered similar topologies that suggest *Cadlinella orniatisma* to be sister to *Hexabranchus sanguineus*. MA is the only phylogenetic estimate that recovered significant support for this relationship (pp = 0.96, bs = 82). *Onchimira cavifera* was recovered nested in the Onchidorididae and sister (CA: pp = 0.98, bs = 82; GA: pp = 0.77, bs = 51; MA: pp = 0.99, bs = 72) to a clade formed by *Knoutsodonta* and *Onchidoris* (CA: pp = 1.00, bs = 86; GA: pp = 1.00, bs = 87; MA: pp = 1.00, bs = 91).

There were only two instances where long branches were evident in the Doridina; *Vayssierea* sp. and a clade formed by *Dendrodoris arborescens, D. fumata, D. guttata* and *D. nigra.* Analysis of nDNA (electronic supplementary material, figure S1) and mtDNA data (electronic supplementary material, figure S2) suggests that the long branches may be an artefact of mtDNA data. The nDNA tree depicts no significantly long branches within the Doridina and recovers a monophyletic *Dendrodoris* clade, which contradicts our mtDNA tree that recovered a polyphyletic *Dendrodoris*.

## 4. Discussion

### 4.1. Resolving the Doridina

Disparity between phylogenetic estimates can confound interpretations and conclusions regarding processes and patterns of lineage diversification. Conflicting estimates are usually consequences of opposing methodologies, which have centred on taxonomic sampling [75–77], molecular markers [12,78], phylogenetic estimates [79–81] and alignment construction [2,3,14,79]. We examined molecular markers, which have been used in varying arrangements, that are most commonly used in nudibranch phylogenetics [21,23,24,49,60–62,82–92].

The large spikes observed in rDNA fragments for our MA and CA are probably a result of highly variable regions or ambiguous sequence calls, which ML is poor at estimating [66], thus overestimating PI towards the tips of their respective tree. Furthermore, PI profiles suggest that information was lost when these regions were excluded from our analyses, and resulted in surprisingly high loss of parsimony informative characters (25%–55%) for rDNA markers. The removal of these variable regions has been shown to negatively affect phylogenetic estimates [9] and may explain why some relationships were not consistently recovered. This supports the position that highly variable loop regions can be vital in resolving some phylogenetic relationships [8]. Unfortunately, we were unable to increase the resolution of the dorid tree by any of our three MSA construction methods. All three PI profiles were fairly consistent in depicting similar curves, and as we have been able to show, these markers are more appropriate for phylogenetic estimates at family- or higher-level classifications.

Another issue we encountered was the potential noise that was incorporated into our estimates by additional taxon sampling. Even though PI profiles suggest these markers were informative at family level, our increased taxonomic sampling may have hindered our ability to recover consistency across analyses. For example, we only recovered a monophyletic Onchidoridoidea in our CA phylogenetic estimate. By contrast, Hallas & Gosliner [62] recovered a mostly resolved monophyletic Onchidoridoidea with significant pp and bs support. Their taxonomic sampling, however, was much more focused and included histone 3 as an additional molecular marker. These contradictory estimates illustrate issues that can result from inappropriate taxonomic sampling [93] and noise incorporated into analyses with inclusion of highly divergent taxa.

In a few instances, however, our expansive taxonomic sampling has illuminated the relationships of some problematic groups, specifically *Aphelodoris,* Cadlinidae, *Cadlinella,* Hexabranchidae and Polyceridae, but in relation to the Onchidoridoidea our estimates contradicted previous highly supported hypotheses. Even though we were able to include *Onchimira cavifera*, it is relevant to state that we were unable to procure other morphologically unique species that may have affected our ability to resolve some family relationships in the Doridina (e.g. *Colga, Goslineria*, *Hoplodoris, Kalinga, Murphydoris, Otinodoris*). These findings illustrate that each phylogenetic query has its own set of challenges and optimal sampling strategy [94], and that the focus for each investigation should be carefully calculated.

### 4.2. Doridina relationships

We were not able to confidently investigate patterns of biogeographical, morphological or chemical evolution due to the lack of resolution at the base of our phylogenetic estimates. The present study, however, offers some consistent new insights into Doridina relationships, in part due to our increased taxonomic sampling.

This work reinforces the conclusion from previous studies that traditional phanerobranch and cryptobranch groupings are not monophyletic [36,59,62]. Even though there was only moderate support in our GA, Cadlinidae does appear to be closely related to at least some members of the Porostomata, despite the ambiguous position of some porostomes such as *Dendrodoris* and *Mandelia*. In addition, we consistently recovered Gymnodorididae and Okadaiidae nested within the Polyceridae, which together are closely related to the Chromodorididae and Hexabranchidae. Unexpectedly, *Cadlinella* was recovered sister to the Hexabranchidae in our phylogenetic estimates. *Cadlinella* was originally included into the Chromodorididae based on morphological similarities [95] and further supported by molecular studies [84,93]. Our broader taxon sampling, however, consistently recovered *Cadlinella* sister to *Hexabranchus* and that both of these taxa, together with the Polyceridae, are closely related to the Chromodorididae. This also is supported by the sperm ultrastructure of *Cadlinella*, which has been shown to be divergent from members of the Chromodorididae [96].

In addition, the yellowish northeastern Pacific species of *Doriopsilla* were thought to represent a species complex of closely related taxa, but this assumption was not tested by including any species from outside the complex, other than the outgroup taxon *D. spauldingi* [97]. In our analysis, we included *D. miniata* from Japan and *D. janaina*, another eastern Pacific species that has divergent colouration. In all three of our analyses, the ‘species complex’ suggested by Hoover *et al*. [97] includes members of two separate lineages, rather than a single radiation. This suggests that these species with yellowish colouration and white spots evolved similar colouration convergently rather than by means of radiation from a single common ancestor.

Lastly, the evolution of the gill pocket is further confounded by the recovery of *Onchimira cavifera* nested within the Onchidorididae. *Onchimira cavifera* was described as having both cryptobranch and phanerobranch characteristics, and hypothesized as a missing link in the current understanding of gill reduction [37,62,98,99]. *Onchimira* possess all the characteristics of a phanerobranch: buccal pump, rectangular rachidian tooth and hooked shaped first lateral tooth, but also possesses a fully formed gill pocket and retractable gill, which is typical of cryptobranch dorids. Surprisingly, *Onchimira* is not closely related to the only other two members of the Onchidorididae that possess similar gill structure to the Cryptobranchia, *Calycidoris* and *Diaphorodoris* [62], but instead nested within the Onchidorididae. Based on our estimates, it is unclear how or under what conditions the gill pocket might have evolved or was lost throughout the Doridina because of the lack of resolution at the base of the tree.

### 4.3. Molecular evolution

It is unclear why there are such large inconsistencies between mtDNA and nDNA phylogenies regarding *Dendrodoris*. To confirm if there was sequencing error, we examined additional specimens of *D. fumata* and *D. nigra* to compare to those on GenBank [61,100]. Surprisingly, all sequences collected were identical. Our inclusion of *D. atromaculata* and *D. denisoni*, however, suggested that there are possible highly divergent regions among mtDNA sequences. We were unable to compare other species of *Dendrodoris* from Hirose *et al.* [100] because they only analysed COI. A complete sampling of *Dendrodoris* is needed to fully comprehend the discrepancies between mtDNA and nDNA sequences. Furthermore, there appears to be no molecular distinction between *Aegires citrinus* and *A. serenae*. Both species are clearly defined by morphological characteristics [101], but both the mtDNA and the nDNA suggest they are in fact the same species. Much like in the Dendrodorididae, it is unclear what molecular mechanisms might have influenced our observations. Further investigations are needed, but are beyond the scope of our data.

## 5. Conclusion

We decided to take an approach that used three common methods used in MSA construction for Nudibranchia phylogenetics. As expected, our findings suggested that MSA methodology affected phylogenetic estimates of the Doridina, especially regarding how we decided to align highly variable rDNA regions. We were able to show that the most commonly sequenced molecular markers for the Nudibranchia lacked the robustness to resolve the base of the dorid tree, and manipulation of highly variable regions affected our ability to recover consistent phylogenetic estimates. This effect, however, is most probably dependent upon the size and scope of the phylogenetic query and amount of missing data. These markers are better suited for higher-level classifications as suggested by our PI profiles. Even though the base of the Doridina was unresolved, family-level classifications were mostly supported across our three analyses, and families that were recovered as non-monophyletic were consistent between alignments. Our analyses suggest that the exclusion of variable regions may have weakened our ability to resolve the base of the Doridina, but previous studies that used much larger datasets have benefited from removing these regions (e.g. [109,110]).

Even though the focus of the present study was to understand MSA construction, our estimates of the Doridina also give a frame of reference for allowing more intensive queries into specific family evolutions. For example, the evolution of caryophyllidia in the Discodorididae, molecular evolution in Aegiridae and Dendrodorididae, or the relationships pertaining to the Polyceridae, Okakaiidae, Gymnodorididae, Hexabranchidae and Chromodorididae, which are some of the most morphologically unique and chemically distinct families.

Nudibranch studies unquestionably suffer from a lack of abundant and diverse molecular markers. Studies have argued that increasing molecular markers could resolve problematic relationships [94,102], but an increase in molecular markers does not resolve issues regarding homology and variable region alignments. Automated filtering protocols allow for MSAs to be easily replicated and eliminate the uncertainty of manual curation of alignments; however, these methods are not without error. In addition, there has been little consensus on the soundest method of increasing signal to resolve phylogenies [6]. Genomic tools, which only have recently been used to investigate nudibranch [103] and larger opisthobranch phylogenetics [104,105], have potential of resolving dorid relationships. However, genomic applications also suffer from alignment and homology issues [106,107]. Phylogenetic resolution of the Doridina can greatly benefit from a genomic approach, but it is important to emphasize the critical role MSAs and homology have on phylogenetic studies. Owing to the varying size and scope of molecular and taxonomic sampling, we strongly recommend the exploration of multiple MSA construction methods that can aid in the selection of an approach that best suits the data.

## Supplementary Material

Bayesian Phylogram of Doridina estimated from nDNA (18s and 28s)

## Supplementary Material

Bayesian Phylogram of Doridina estimated from mtDNA (16s and COI)

## Data accessibility

Supporting files are accessible in the electronic supplementary material. Sequence alignments have been deposited in TreeBASE (http://purl.org/phylo/treebase/phylows/study/TB2:S20396). Finally, DNA sequences (listed in table 1) have been uploaded to GenBank accession ranges: 16S(MF958278-MF958318); 18S(MF958319-MF958362); 28S(MF958363-MF958409); COI(MF958411-MF958446).

## References

[1] Morrison DA, Morgan MJ, Kelchner SA 2015 Molecular homology and multiple-sequence alignment: an analysis of concepts and practice. Aust. Syst. Bot. 28, 46–62. (doi:10.1071/SB15001)

[2] Morgan MJ, Kelchner SA 2010 Inference of molecular homology and sequence alignment by direct optimization. Mol. Phylogenet. Evol. 56, 305–311. (doi:10.1016/j.ympev.2010.03.032) 20363340 10.1016/j.ympev.2010.03.032

[3] Morrison DA 2015 Is sequence alignment an art or a science? Syst. Bot. 40, 14–26. (doi:10.1600/036364415X686305)

[4] Patterson C 1988 Homology in classical and molecular biology. Mol. Biol. Evol. 5, 603–625. 3065587 10.1093/oxfordjournals.molbev.a040523

[5] Smith TF, Waterman MS 1981 Identification of common molecular subsequences. J. Mol. Biol. 147, 195–197. (doi:10.1016/0022-2836(81)90087-5) 7265238 10.1016/0022-2836(81)90087-5

[6] Tan G, Muffato M, Ledergerber C, Herrero J, Goldman N, Gil M, Dessimoz C 2015 Current methods for automated filtering of multiple sequence alignments frequently worsen single-gene phylogenetic inference. Syst. Biol. 64, 778–791. (doi:10.1093/sysbio/syv033) 26031838 10.1093/sysbio/syv033 PMC4538881

[7] Wheeler WC 1996 Optimization alignment: the end of multiple sequence alignment in phylogenetics? Cladistics 12, 1–9. (doi:10.1006/clad.1996.0001)

[8] Dessimoz C, Gil M 2010 Phylogenetic assessment of alignments reveals neglected tree signal in gaps. Genome Biol. 11, R37 (doi:10.1186/gb-2010-11-4-r37) 20370897 10.1186/gb-2010-11-4-r37 PMC2884540

[9] Townsend JP 2007 Profiling phylogenetic informativeness. Syst. Biol. 56, 222–231. (doi:10.1080/10635150701311362) 17464879 10.1080/10635150701311362

[10] Morrison DA 2009 Why would phylogeneticists ignore computerized sequence alignment? Syst. Biol. 58, 150–158. (doi:10.1093/sysbio/syp009) 20525575 10.1093/sysbio/syp009

[11] Talavera G, Castresana J 2007 Improvement of phylogenies after removing divergent and ambiguously aligned blocks from protein sequence alignments. Syst. Biol. 56, 564–577. (doi:10.1080/10635150701472164) 17654362 10.1080/10635150701472164

[12] Brocchieri L 2001 Phylogenetic inferences from molecular sequences: review and critique. Theor. Popul. Biol. 59, 27–40. (doi:10.1006/tpbi.2000.1485) 11243926 10.1006/tpbi.2000.1485

[13] Aagesen L 2004 The information content of an ambiguously alignable region, a case study of the *trnL* intron from the Rhamnaceae. Org. Divers. Evol. 4, 35–49. (doi:10.1016/j.ode.2003.11.003)

[14] Lee MSY 2001 Unalignable sequences and molecular evolution. Trends Ecol. Evol. 16, 681–685. (doi:10.1016/S0169-5347(01)02313-8)

[15] Castresana J 2000 Selection of conserved blocks from multiple alignments for their use in phylogenetic analysis. Mol. Biol. Evol. 17, 540–552. (doi:10.1093/oxfordjournals.molbev.a026334) 10742046 10.1093/oxfordjournals.molbev.a026334

[16] Grundy WN, Naylor GJP 1999 Phylogenetic inference from conserved sites alignments. J. Exp. Zool. 285, 128–139. (doi:10.1002/(SICI)1097-010X(19990815)285:2<128::AID-JEZ5>3.0.CO;2-C) 10440724

[17] Löytynoja A, Milinkovitch MC 2001 SOAP, cleaning multiple alignments from unstable blocks. Bioinformatics 17, 573–574. (doi:10.1093/bioinformatics/bth449) 11395440 10.1093/bioinformatics/17.6.573

[18] Swofford DL, Olsen GJ, Waddell PJ, Hillis DM 1996 Phylogenetic inference. In Molecular systematics (eds Hillis DM, Moritz C, Mable BK), pp. 407–514. Sunderland, MA: Sinauer.

[19] Wheeler WC 1995 Sequence alignment, parameter sensitivity, and the phylogenetic analysis of molecular data. Syst. Biol. 44, 321–331. (doi:10.1093/sysbio/44.3.321)

[20] Doyle JJ, Davis JI 1998 Homology in molecular phylogenetics: a parsimony perspective. In Molecular systematics of plants II (eds DE Soltis, PS Soltis, JJ Doyle), pp. 101–131. Berlin, Germany: Springer US.

[21] Vonnemann V, Schrödl M, Klussmann-Kolb A, Wägele H 2005 Reconstruction of the phylogeny of the Opisthobranchia (Mollusca: Gastropoda) by means of 18s and 28s rRNA gene sequences. J. Molluscan Stud. 71, 113–125. (doi:10.1093/mollus/eyi014)

[22] Pola M, Gosliner TM 2010 The first molecular phylogeny of cladobranchian opisthobranchs (Mollusca, Gastropoda, Nudibranchia). Mol. Phylogenet. Evol. 56, 931–941. (doi:10.1016/j.ympev.2010.05.003) 20460158 10.1016/j.ympev.2010.05.003

[23] Göbbeler K, Klussmann-Kolb A 2010 Out of Antarctica?—new insights into the phylogeny and biogeography of the Pleurobranchomorpha (Mollusca, Gastropoda). Mol. Phylogenet. Evol. 55, 996–1007. (doi:10.1016/j.ympev.2009.11.027) 19995612 10.1016/j.ympev.2009.11.027

[24] Dinapoli A, Klussmann-Kolb A 2010 The long way to diversity—phylogeny and evolution of the Heterobranchia (Mollusca: Gastropoda). Mol. Phylogenet. Evol. 55, 60–76. (doi:10.1016/j.ympev.2009.09.019) 19778622 10.1016/j.ympev.2009.09.019

[25] Schrödl M, Jörger KM, Klussmann-Kolb A, Wilson NG 2011 Bye bye ‘opisthobranchia’! A review on the contribution of mesopsammic sea slugs to euthyneuran systematics. Thalassas 27, 101–112.

[26] Wägele H, Klussmann-Kolb A, Verbeek E, Schrödl M 2014 Flashback and foreshadowing—a review of the taxon Opisthobranchia. Org. Divers. Evol. 14, 133–149. (doi:10.1007/s13127-013-0151-5)

[27] Faulkner DJ, Ghiselin MT 1983 Chemical defense and evolutionary ecology of dorid nudibranchs and some other opisthobranch gastropods. Mar. Ecol. Prog. Ser. 13, 295–301. (doi:10.3354/meps013295)

[28] Cimino G, De Rosa S, De Stefano S, Sodano G, Villani G 1983 Dorid nudibranch elaborates its own chemical defense. Science 219, 1237–1238. (doi:10.1126/science.219.4589.1237) 17771309 10.1126/science.219.4589.1237

[29] Parlik JR, Kernan MR, Molinski TF, Harper MK, Faulkner DJ 1988 Defensive chemicals of the Spanish dancer nudibranch *Hexabranchus sanguineus* and its egg ribbons: macrolides derived from a sponge diet. J. Exp. Mar. Bio. Ecol. 119, 99–109. (doi:10.1016/0022-0981(88)90225-0)

[30] Faulkner DJ, Molinski TF, Andersen RJ, Dumdei EJ, De Silva ED 1990 Geographical variation in defensive chemicals from pacific coast dorid nudibranchs and some related marine molluscs. Comp. Biochem. Physiol. Part C, Comp. 97, 233–240. (doi:10.1016/0742-8413(90)90133-T)

[31] Cimino G, Ghiselin MT 1999 Chemical defense and evolutionary trends in biosynthetic capacity among dorid nudibranchs (Mollusca: Gastropoda: Opisthobranchia). Chemoecology 9, 187–207. (doi:10.1007/s000490050052)

[32] Wilson NG, Maschek JA, Baker BJ 2013 A species flock driven by predation? Secondary metabolites support diversification of slugs in Antarctica. PLoS ONE 8, 1–7. (doi:10.1371/journal.pone.0080277) 10.1371/journal.pone.0080277 PMC3841181 24303002

[33] Hirayama Y, Katavic PL, White AM, Pierens GK, Lambert LK, Winters AE, Kigoshi H, Kita M, Garson MJ 2016 New cytotoxic norditerpenes from the Australian nudibranchs *Goniobranchus splendidus* and *Goniobranchus daphne*. Aust. J. Chem. 69, 136–144. (doi:10.1071/CH15203)

[34] Gosliner TM, Valdés ÁA 1999 Phylogeny of the radula-less dorids (Mollusca, Nudibranchia), with the description of a new genus and a new family. Zool. Scr. 28, 315–360. (doi:10.1046/j.1463-6409.1999.00014.x)

[35] Wilson NG, Healy JM 2002 Comparative sperm ultrastructure in five genera of the nudibranch family Chromodorididae (Gastropoda: Opisthobranchia). J. Molluscan Stud. 68, 133–145. (doi:10.1093/mollus/68.2.133) 12011239 10.1093/mollus/68.2.133

[36] Valdés ÁA 2002 A phylogenetic analysis and systematic revision of the cryptobranch dorids (Mollusca, Nudibranchia, Anthobranchia). Zool. Scr. 136, 535–636.

[37] Martynov AV, Schrödl M 2011 Phylogeny and evolution of corambid nudibranchs (Mollusca: Gastropoda). Zool. J. Linn. Soc. 163, 585–604. (doi:10.1111/j.1096-3642.2011.00720.x)

[38] Fahey SJ, Healy JM 2003 Sperm ultrastructure in the nudibranch genus *Halgerda* with reference to other Discodorididae and to Chromodorididae (Mollusca: Opisthobranchia). J. Morphol. 257, 9–21. (doi:10.1002/jmor.10086) 12740892 10.1002/jmor.10086

[39] Valdés ÁA 2004 Morphology of the penial hooks and vaginal cuticular lining of some dorid nudibranchs (Mollusca, Opisthobranchia). Am. Malacol. Bull. 18, 49–53.

[40] Dayrat B 2010 A monographic revision of basal discodorid sea slugs (Mollusca: Gastropoda: Nudibranchia: Doridina). Proc. Calif. Acad. Sci. 61, 1–403.

[41] Martynov AV 2011 From ‘tree-thinking’ to ‘cycle-thinking’: ontogenetic systematics of nudibranch molluscs. Thalassas 27, 193–224.

[42] Martynov AV, Brenzinger B, Hooker Y, Schrödl M 2010 3D-anatomy of a new tropical Peruvian nudibranch gastropod species, *Corambe mancorensis*, and novel hypotheses on dorid gill ontogeny and evolution. J. Molluscan Stud. 77, 129–141. (doi:10.1093/mollus/eyq047)

[43] Rudman WB 1991 Purpose in pattern—the evolution of color in chromodorid Nudibranchs. J. Molluscan Stud. 57, 5–21. (doi:10.1093/mollus/57.Supplement_Part_4.5)

[44] Valdés ÁA 2001 Depth-related adaptations, speciation processes and evolution of color in the genus *Phyllidiopsis* (Mollusca: Nudibranchia). Mar. Biol. 139, 485–496. (doi:10.1007/s002270100596)

[45] Haber M et al. 2010 Coloration and defense in the nudibranch gastropod *Hypselodoris fontandraui*. Biol. Bull. 218, 181–188. (doi:10.1086/BBLv218n2p181) 20413794 10.1086/BBLv218n2p181

[46] Johnson RF, Gosliner TM. 2012 Traditional taxonomic groupings mask evolutionary history: a molecular phylogeny and new classification of the chromodorid nudibranchs. PLoS ONE 7, e33479 (doi:10.1371/journal.pone.0033479) 22506002 10.1371/journal.pone.0033479 PMC3323602

[47] Valdés ÁA, Ornelas-Gatdula E, Dupont A 2013 Color pattern variation in a shallow-water species of opisthobranch mollusc. Biol. Bull. 224, 35–46. (doi:10.1086/BBLv224n1p35) 23493507 10.1086/BBLv224n1p35

[48] Padula V, Bahia J, Stöger I, Camacho-García Y, Malaquias MAE, Cervera JL, Schrödl M 2016 A test of color-based taxonomy in nudibranchs: molecular phylogeny and species delimitation of the *Felimida clenchi* (Mollusca: Chromodorididae) species complex. Mol. Phylogenet. Evol. 103, 215–229. (doi:10.1016/j.ympev.2016.07.019) 27444708 10.1016/j.ympev.2016.07.019

[49] Hallas JM, Simison WB, Gosliner TM 2016 Dating and biogeographical patterns in the sea slug genus *Acanthodoris* Gray, 1850 (Mollusca, Gastropoda, Nudibranchia). Mol. Phylogenet. Evol. 97, 19–31. (doi:10.1016/j.ympev.2015.12.018) 26752594 10.1016/j.ympev.2015.12.018

[50] Lambert WJ 2013 Population biology of an intertidal dorid nudibranch (*Onchidoris muricata*) in the Southern Gulf of Maine, USA: changes in phenology due to an invasive prey? Am. Malacol. Bull. 31, 17–23. (doi:10.4003/006.031.0109)

[51] Lindsay T, Valdés ÁA 2016 The model organism *Hermissenda crassicornis* (Gastropoda: Heterobranchia) is a species complex. PLoS ONE 11, e0154265 (doi:10.1371/journal.pone.0154265) 27105319 10.1371/journal.pone.0154265 PMC4841509

[52] Todd CD, Lambert WJ, Thorpe JP 1998 The genetic structure of intertidal populations of two species of nudibranch molluscs with planktotrophic and pelagic lecithotrophic larval stages: are pelagic larvae ‘for’ dispersal? J. Exp. Mar. Bio. Ecol. 228, 1–28. (doi:10.1016/S0022-0981(98)00005-7)

[53] Jones HL, Todd CD, Lambert WJ 1996 Intraspecific variation in embryonic and larval traits of the dorid nudibranch mollusc *Adalaria proxima* (Alder and Hancock) around the northern coasts of the British Isles. J. Exp. Mar. Bio. Ecol. 202, 29–47. (doi:10.1016/0022-0981(96)00029-9)

[54] Lambert WJ, Todd CD, Hardege JD 1997 Partial characterization and biological activity of a metamorphic inducer of the dorid nudibranch *Adalaria proxima* (Gastropoda: Nudibranchia). Invertebr. Biol. 116, 71–81. (doi:10.2307/3226971)

[55] Goddard JHR 2008 Developmental mode in opisthobranch Molluscs from the tropical Eastern Pacific Ocean. The Veliger 50, 83–96.

[56] Goddard JHR 2004 Developmental mode in benthic opisthobranch molluscs from the northeast Pacific Ocean: feeding in a sea of plenty. Can. J. Zool. 82, 1954–1968. (doi:10.1016/j.tree.2011.11.016)

[57] Goddard JHR, Green B. 2013 Developmental mode in opisthobranch molluscs from the northeast Pacific Ocean: additional species from Southern California and supplemental data. Bull. South. Calif. Acad. Sci. 112 49–62 (doi:10.3160/0038-3872-112.2.49)

[58] Valdés ÁA 2004 Phylogeography and phyloecology of dorid nudibranchs (Mollusca, Gastropoda). Biol. J. Linn. Soc. 83, 551–559. (doi:10.1111/j.1095-8312.2004.00413.x)

[59] Wägele H, Willan RC 2000 Phylogeny of the Nudibranchia. Zool. J. Linn. Soc. 130, 83–181. (doi:10.1006/zjls)

[60] Wollscheid-Lengeling E, Wägele H 1999 Initial results on the molecular phylogeny of the Nudibranchia (Gastropoda, Opisthobranchia) based on 18S rDNA data. Mol. Phylogenet. Evol. 13, 215–226. (doi:10.1006/mpev.1999.0664) 10603252 10.1006/mpev.1999.0664

[61] Wollscheid-Lengeling E, Boore JL, Brown W, Wägele H 2001 The phylogeny of Nudibranchia (Opisthobranchia, Gastropoda, Mollusca) reconstructed by three molecular markers. Org. Divers. Evol. 1, 241–256. (doi:10.1078/1439-6092-00022)

[62] Hallas JM, Gosliner TM 2015 Family matters: the first molecular phylogeny of the Onchidorididae Gray, 1827 (Mollusca, Gastropoda, Nudibranchia). Mol. Phylogenet. Evol. 88, 16–27. (doi:10.1016/j.ympev.2015.03.015) 25837732 10.1016/j.ympev.2015.03.015

[63] Kearse M et al. 2012 Geneious basic: an integrated and extendable desktop software platform for the organization and analysis of sequence data. Bioinformatics 28, 1647–1649. (doi:10.1093/bioinformatics/bts199) 22543367 10.1093/bioinformatics/bts199 PMC3371832

[64] Hall T 1999 BioEdit: a user-friendly biological sequence alignment editor and analysis program for windows 95/98/NT. Nucleic Acids Symp. Ser. 41, 95–98. (doi:citeulike-article-id:691774)

[65] Katoh K, Standley DM 2013 MAFFT multiple sequence alignment software version 7: improvements in performance and usability. Mol. Biol. Evol. 30, 772–780. (doi:10.1093/molbev/mst010) 23329690 10.1093/molbev/mst010 PMC3603318

[66] López-Giráldez F, Townsend JP. 2011 PhyDesign: an online application for profiling phylogenetic informativeness. BMC Evol. Biol. 11, 152 (doi:10.1186/1471-2148-11-152) 21627831 10.1186/1471-2148-11-152 PMC3124428

[67] Kosakovsky Pond SL, Frost SDW, Muse SV 2005 HyPhy: hypothesis testing using phylogenies. Bioinformatics 21, 676–679. (doi:10.1093/bioinformatics/bti079) 15509596 10.1093/bioinformatics/bti079

[68] Maddison WP, Maddison DR. (2017). http://mesquiteproject.org.

[69] Moeller AH, Townsend JP 2011 Phylogenetic informativeness profiling of 12 genes for 28 vertebrate taxa without divergence dates. Mol. Phylogenet. Evol. 60, 271–272. (doi:10.1016/j.ympev.2011.04.023) 21558010 10.1016/j.ympev.2011.04.023

[70] Lanfear R, Calcott B, Ho SYW, Guindon S 2012 PartitionFinder: combined selection of partitioning schemes and substitution models for phylogenetic analyses. Mol. Biol. Evol. 29, 1695–1701. (doi:10.1093/molbev/mss020) 22319168 10.1093/molbev/mss020

[71] Ronquist F, Huelsenbeck JP 2003 MrBayes 3: Bayesian phylogenetic inference under mixed models. Bioinformatics 19, 1572–1574. (doi:10.1093/bioinformatics/btg180) 12912839 10.1093/bioinformatics/btg180

[72] Rambaut A, Suchard MA, Xie D, Drummond AJ 2014 Tracer v1.5. See http://tree.bio.ed.ac.uk/software/tracer/.

[73] Stamatakis A 2006 RAxML-VI-HPC: maximum likelihood-based phylogenetic analyses with thousands of taxa and mixed models. Bioinformatics 22, 2688–2690. (doi:10.1093/bioinformatics/btl446) 16928733 10.1093/bioinformatics/btl446

[74] Hillis DM, Bull JJ 1993 An empirical test of bootstrapping as a method for assessing confidence in phylogenetic analysis. Syst. Biol. 42, 182–192. (doi:10.1093/sysbio/42.2.182)

[75] Hillis DM 1998 Taxonomic sampling, phylogenetic accuracy, and investigator bias. Syst. Biol. 47, 3–8. (doi:10.1080/106351598260987) 12064238 10.1080/106351598260987

[76] Zwickl DJ, Hillis DM 2002 Increased taxon sampling greatly reduces phylogenetic error. Syst. Biol. 51, 588–598. (doi:10.1080/10635150290102339) 12228001 10.1080/10635150290102339

[77] Rosenberg MS, Kumar S 2001 Incomplete taxon sampling is not a problem for phylogenetic inference. Proc. Natl Acad. Sci. USA 98, 10 751–10 756. (doi:10.1073/pnas.191248498) 10.1073/pnas.191248498 PMC58547 11526218

[78] Philippe H, Brinkmann H, Lavrov DV, Littlewood DTJ, Manuel M, Wörheide G, Baurain D 2011 Resolving difficult phylogenetic questions: why more sequences are not enough. PLoS Biol. 9, e1000602 (doi:10.1371/journal.pbio.1000602) 21423652 10.1371/journal.pbio.1000602 PMC3057953

[79] Kjer KM, Gillespie JJ, Ober KA 2007 Opinions on multiple sequence alignment, and an empirical comparison of repeatability and accuracy between POY and structural alignment. Syst. Biol. 56, 133–146. (doi:10.1080/10635150601156305) 17366144 10.1080/10635150601156305

[80] Huelsenbeck JP, Larget B, Miller RE, Ronquist F 2002 Potential applications and pitfalls of Bayesian inference of phylogeny. Syst. Biol. 51, 673–688. (doi:10.1080/10635150290102366) 12396583 10.1080/10635150290102366

[81] Douady CJ, Delsuc F, Boucher Y, Doolittle WF, Douzery EJP 2003 Comparison of Bayesian and maximum likelihood bootstrap measures of phylogenetic reliability. Mol. Biol. Evol. 20, 248–254. (doi:10.1093/molbev/msg042) 12598692 10.1093/molbev/msg042

[82] Grande C, Templado J, Cervera JL, Zardoya R 2004 Phylogenetic relationships among Opisthobranchia (Mollusca: Gastropoda) based on mitochondrial *cox 1*, *trnV*, and *rrnL* genes. Mol. Phylogenet. Evol. 33, 378–388. (doi:10.1016/j.ympev.2004.06.008) 15336672 10.1016/j.ympev.2004.06.008

[83] Pola M, Cervera JL, Gosliner TM 2007 Phylogenetic relationships of Nembrothinae (Mollusca: Doridacea: Polyceridae) inferred from morphology and mitochondrial DNA. Mol. Phylogenet. Evol. 43, 726–742. (doi:10.1016/j.ympev.2007.02.003) 17470399 10.1016/j.ympev.2007.02.003

[84] Turner LM, Wilson NG 2008 Polyphyly across oceans: a molecular phylogeny of the Chromodorididae (Mollusca, Nudibranchia). Zool. Scr. 37, 23–42. (doi:10.1111/j.1463-6409.2007.00310.x)

[85] Stout CC, Pola M, Valdés ÁA 2010 Phylogenetic analysis of *Dendronotus* nudibranchs with emphasis on northeastern Pacific species. J. Molluscan Stud. 76, 367–375. (doi:10.1093/mollus/eyq022)

[86] Carmona L, Pola M, Gosliner TM, Cervera JL 2013 A tale that morphology fails to tell: a molecular phylogeny of Aeolidiidae (Aeolidida, Nudibranchia, Gastropoda). PLoS ONE 8, e63000 (doi:10.1371/journal.pone.0063000) 23658794 10.1371/journal.pone.0063000 PMC3642091

[87] Churchill CKC, Valdés ÁA, Ó Foighil D 2014 Molecular and morphological systematics of neustonic nudibranchs (Mollusca: Gastropoda: Glaucidae: Glaucus), with descriptions of three new cryptic species. Invertebr. Syst. 28, 174 (doi:10.1071/IS13038)

[88] Hulett RE, Mahguib J, Gosliner TM, Valdés ÁA 2015 Molecular evaluation of the phylogenetic position of the enigmatic species *Trivettea papalotla* (Bertsch) (Mollusca: Nudibranchia). Invertebr. Syst. 29, 215–222. (doi:10.1071/is15002)

[89] Shipman C, Gosliner T 2015 Molecular and morphological systematics of *Doto* Oken, 1851 (Gastropoda: Heterobranchia), with descriptions of five new species and a new genus. Zootaxa 3973, 057–101. (doi:10.11646/zootaxa.3973.1.2) 10.11646/zootaxa.3973.1.2 26249713

[90] Furfaro G, Picton B, Martynov AV, Mariottini P 2016 *Diaphorodoris alba* Portmann & Sandmeier, 1960 is a valid species: molecular and morphological comparison with *D. luteocincta* (M. Sars, 1870) (Gastropoda: Nudibranchia). Zootaxa 4193, 304–316. (doi:10.11646/zootaxa.4193.2.6) 10.11646/zootaxa.4193.2.6 27988719

[91] Combosch DJ et al. 2017 A family-level tree of life for bivalves based on a Sanger-sequencing approach. Mol. Phylogenet. Evol. 107, 191–208. (doi:10.1016/j.ympev.2016.11.003) 27840226 10.1016/j.ympev.2016.11.003

[92] Cella K, Carmona L, Ekimova I, Chichvarkhin A, Schepetov D, Gosliner TM 2016 A radical solution: the phylogeny of the nudibranch family Fionidae. PLoS ONE 11, e0167800 (doi:10.1371/journal.pone.0167800) 27977703 10.1371/journal.pone.0167800 PMC5158052

[93] Johnson RF 2010 Breaking family ties: taxon sampling and molecular phylogeny of chromodorid nudibranchs (Mollusca, Gastropoda). Zool. Scr. 40, 137–157. (doi:10.1111/j.1463-6409.2010.00457.x)

[94] Rokas A, Carroll SB 2005 More genes or more taxa? The relative contribution of gene number and taxon number to phylogenetic accuracy. Mol. Biol. Evol. 22, 1337–1344. (doi:10.1093/molbev/msi121) 15746014 10.1093/molbev/msi121

[95] Rudman WB 1984 The Chromodorididae (Opisthobranchia: Mollusca) of the Indo-West Pacific: a review of the genera. Zool. J. Linn. Soc. 81, 115–273. (doi:10.1111/j.1096-3642.1984.tb01174.x)

[96] Wilson NG, Healy JM 2002 Is *Cadlinella ornatissima* a chromodorid? Sperm ultrastructure in an enigmatic nudibranch (Opisthobranchia, Mollusca). Invertebr. Reprod. Dev. 42, 179–188. (doi:10.1080/07924259.2002.9652774)

[97] Hoover C, Lindsay T, Goddard JHR, Valdés ÁA 2015 Seeing double: pseudocryptic diversity in the *Doriopsilla albopunctata-Doriopsilla gemela* species complex of the north-eastern Pacific. Zool. Scr. 44, 612–631. (doi:10.1111/zsc.12123)

[98] Martynov AV, Korshunova T, Sanamyan N, Sanamyan K 2009 Description of the first cryptobranch onchidoridid *Onchimira cavifera* gen. et sp. nov., and of three new species of the genera *Adalaria* Bergh, 1879 and *Onchidoris* Blainville, 1816 (Nudibranchia: Onchidorididae) from Kamchatka waters. Zootaxa 43, 1–43.

[99] Martynov AV, Korshunova T. 2011 Opisthobranch molluscs of the seas of Russia. A colour guide to their taxonomy and biology. Moscow, Russia: Fiton+.

[100] Hirose M, Hirose E, Kiyomoto M 2015 Identification of five species of *Dendrodoris* (Mollusca: Nudibranchia) from Japan, using DNA barcode and larval characters. Mar. Biodivers. 45, 769–780. (doi:10.1007/s12526-014-0288-2)

[101] Fahey SJ, Gosliner TM 2004 A phylogenetic analysis of the Aegiridae Fischer, 1883 (Mollusca, Nudibranchia, Phanerobranchia) with descriptions of eight new species and a reassessment of phanerobranch relationships. Proc. Calif. Acad. Sci. 55, 613–689.

[109] Delsuc F, Brinkmann H, Philippe H 2005 Phylogenomics and the reconstruction of the tree of life. Nat. Rev. Genet. 6, 361–375. (doi:10.1038/nrg1603) 15861208 10.1038/nrg1603

[110] Mitros T, Richards GS, Conaco C, Dacre M, Degnan SM, Oakley TH, Plachetzki DC, Zhai Y 2010 The *Amphimedon queenslandica* genome and the evolution of animal complexity. Nature 466, 720–726. (doi:10.1038/nature09201). 20686567 10.1038/nature09201 PMC3130542

[102] Doyle VP, Young RE, Naylor GJP, Brown JM 2015 Can we identify genes with increased phylogenetic reliability? Syst. Biol. 64, 824–837. (doi:10.1093/sysbio/syv041) 26099258 10.1093/sysbio/syv041

[103] Goodheart JA, Bazinet AL, Collins AG, Cummings MP 2015 Relationships within Cladobranchia (Gastropoda: Nudibranchia) based on RNA-Seq data: an initial investigation. R. Soc. open. sci. 2, 150196 (doi:10.1098/rsos.150196) 26473045 10.1098/rsos.150196 PMC4593679

[104] Medina M, Lal S, Vallès Y, Takaoka TL, Dayrat B, Boore JL, Gosliner TM 2011 Crawling through time: transition of snails to slugs dating back to the Paleozoic, based on mitochondrial phylogenomics. Mar. Genomics 4, 51–59. (doi:10.1016/j.margen.2010.12.006) 21429465 10.1016/j.margen.2010.12.006

[105] Kocot KM, Halanych KM, Krug PJ 2013 Phylogenomics supports Panpulmonata: Opisthobranch paraphyly and key evolutionary steps in a major radiation of gastropod molluscs. Mol. Phylogenet. Evol. 69, 764–771. (doi:10.1016/j.ympev.2013.07.001) 23850501 10.1016/j.ympev.2013.07.001

[106] Nater A, Burri R, Kawakami T, Smeds L, Ellegren H 2015 Resolving evolutionary relationships in closely related species with whole-genome sequencing data. Syst. Biol. 46, 1–54. (doi:10.1093/sysbio/syv045) 10.1093/sysbio/syv045 PMC4604831 26187295

[107] Leaché AD, Banbury BL, Felsenstein J, De Oca ANM, Stamatakis A 2015 Short tree, long tree, right tree, wrong tree: new acquisition bias corrections for inferring SNP phylogenies. Syst. Biol. 64, 1032–1047. (doi:10.1093/sysbio/syv053) 26227865 10.1093/sysbio/syv053 PMC4604835

