# Supplementary figures and images for: Aligning evidence: concerns regarding multiple sequence alignments in estimating the phylogeny of the Nudibranchia suborder Doridina
Source: R Soc Open Sci. 2017 Oct 25;4(10):171095. doi: 10.1098/rsos.171095 (PMC5666284; doi:10.1098/rsos.171095)

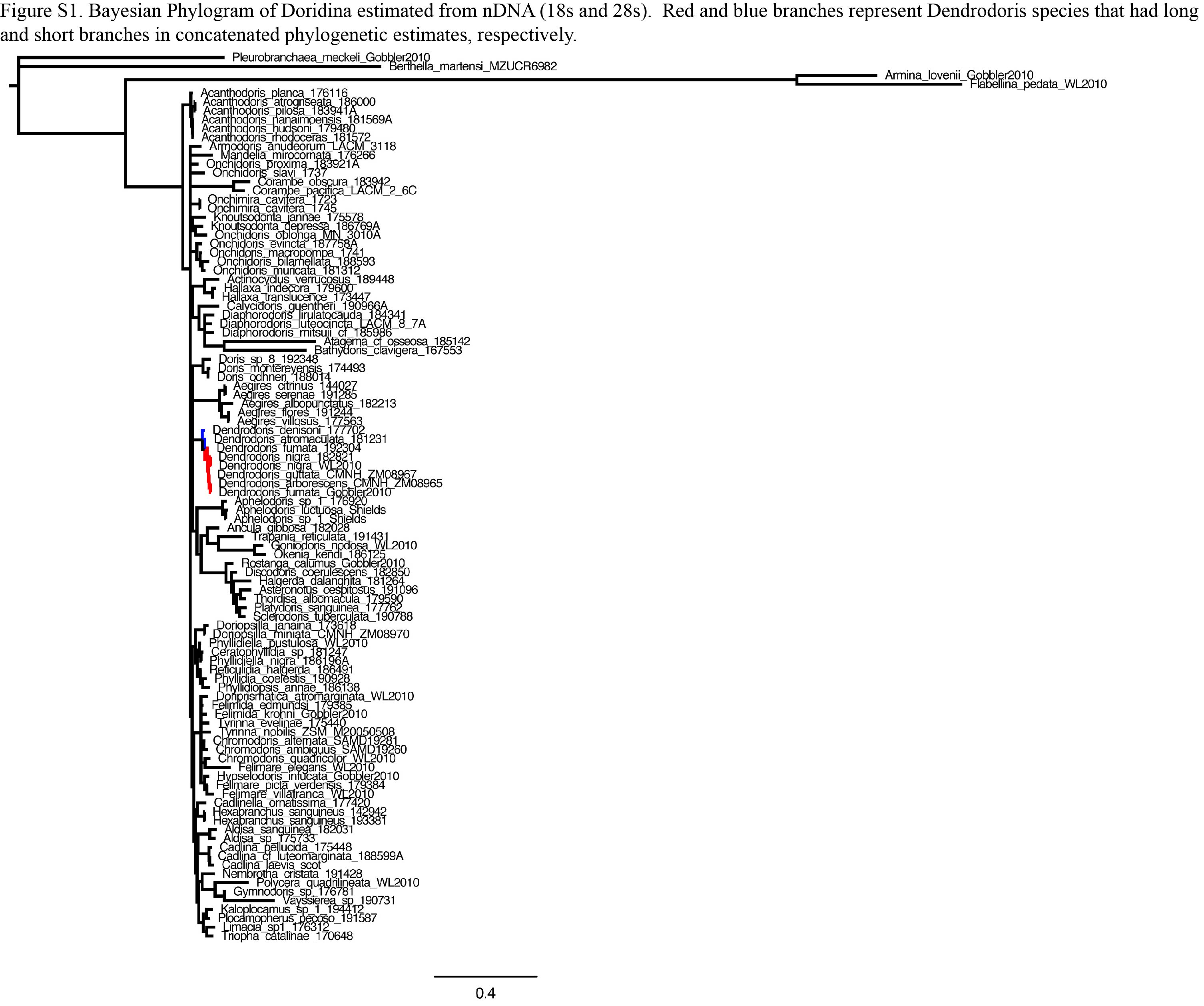

Supplement: Bayesian Phylogram of Doridina estimated from nDNA (18s and 28s) [file rsos171095supp1.jpg]

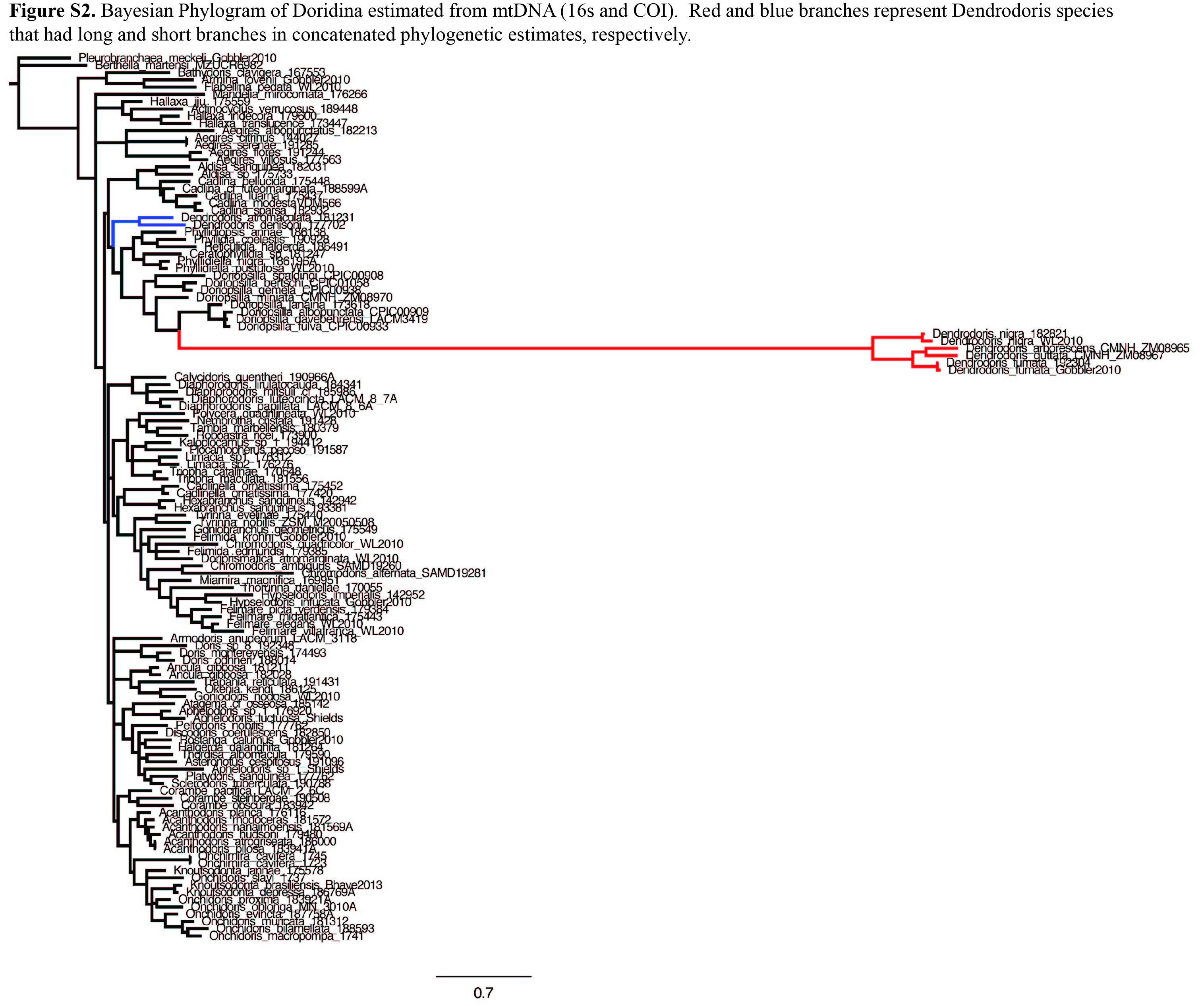

Supplement: Bayesian Phylogram of Doridina estimated from mtDNA (16s and COI) [file rsos171095supp2.jpg]
